# Quercetin attenuates reduced uterine perfusion pressure -induced hypertension in pregnant rats through regulation of endothelin-1 and endothelin-1 type A receptor

**DOI:** 10.1186/s12944-020-01357-w

**Published:** 2020-08-05

**Authors:** Xia Sun, Shuping Zhang, Haitao Song

**Affiliations:** Department of obstetrics, Qingdao Municipal Hospital, No. 5 Donghai Road, Qingdao, 266071 Shandong China

**Keywords:** Quercetin, Reduced uterine perfusion pressure, Hypertension, Pregnant rats, Pharmacology

## Abstract

**Background:**

Quercetin was reported to be crucial for a broad range of activities, including attenuating inflammation, platelet aggregation, capillary permeability, and lipid peroxidation. However, the effect of quercetin in hypertension during pregnancy, was not fully understood.

**Methods:**

The model of hypertension in pregnancy was established in rats by reduced uterine perfusion pressure (RUPP). Quercetin was administrated by gavage. Systolic blood pressure (SBP) and diastolic blood pressure (DBP) were measured using the CODA 6 BP system. Plasma concentrations of Endothelin-1 (ET-1), soluble fms-like tyrosine kinase-1 (sFlt-1), and vascular endothelial growth factor (VEGF) were detected using enzyme-linked immunosorbent assay kits. The mRNA and protein levels of ET-1 and endothelin-1 type A receptor (ET_A_R) were determined by RT-PCR and Western blotting. The ET_A_R antagonist BQ-123 was performed by osmotic minipumps.

**Results:**

In RUPP induced rats, quercetin treatment decreased SBP and DBP, fetal resorptions percentage, plasma ET-1 and sFlt-1 concentrations, ET-1 and ET_A_R levels, but increased fetal body weight and VEGF expression. BQ-123 administration attenuated SBP and DBP, suppressed fatal resorptions percentage, and increased fetal body weight of RUPP rats.

**Conclusion:**

Quercetin attenuates RUPP induced hypertension in pregnant rats through the regulation of ET-1 and ET_A_R.

## Introduction

Hypertension in pregnancy is the disease that developed in mother gestation of 20 weeks or longer and is one of the leading causes of morbidity and mortality for mother and baby around the world [1, 2]. Hypertension in pregnancy is characterized by sudden hypertension development with over 140/90 blood pressure, proteinuria, elevated liver enzymes, hemolytic anemia, and low platelet count [3, 4]. The disorder during hypertension in pregnancy begins with a normal pregnant process and will finally cause severe maternal and fetal health problems [5, 6]. Moreover, when comparing with normal pregnant women, women with history of hypertension in pregnancy have a higher risk to develop higher blood pressure, long term endothelial dysfunction, and low-density lipoprotein cholesterol [7]. Hypertension in pregnancy commonly leads to complications in the brain, heart, and kidneys [7]. Early diagnosis is crucial for the therapy of hypertension in pregnancy. However, the mechanism that regulated the pathogenesis of hypertension in pregnancy in not totally understood, which limits the options for potential treatment [8].

Quercetin could be widely found in vegetables, fruits, and soybeans [9]. Various studies reported the effect of quercetin in anti-hypertension both in human and animal models. Daily intake of 150–730 mg quercetin for 4 to 10 weeks showed the anti-hypertensive effect in human. For stage 1 hypertensive patients, a high dose (730 mg daily) of quercetin intake significantly suppressed systolic blood pressures (SBP) and diastolic blood pressures (DBP), while high dose quercetin showed no effect for prehypertension patients [10]. In patients with metabolic syndrome, Daily intake of 150 mg quercetin for 5 weeks suppressed SBP [11]. In a hypertensive rat model, 10^− 5^ M quercetin treatment increased acetylcholine-induced vascular relaxation [12], demonstrating that quercetin suppressed blood pressure by attenuating the blood vessel elastance. However, the effect of quercetin on hypertension in pregnancy remains unclear and the mechanism is still waiting for exploration.

In this study, the reduced uterine perfusion pressure (RUPP) rat model [13] was used to explore the effect of quercetin in hypertension during pregnancy. In RUPP rats, quercetin treatment decreased blood pressure, and fetal resorptions percentage, and increased fetal body weight. Mechanically, quercetin treatment inhibited the Endothelin-1 (ET-1) / endothelin-1 type A receptor (ET_A_R) pathway. These results supply evidence that quercetin may be used for the treatment of hypertension in pregnancy in the clinic.

## Methods

### PUPP rat model

Sprague-Dawley female rats (210–240 g) and male rats (260–300 g) procured from HFK Bioscience (Beijing, China), aged 10 weeks, were monitored in sterile cages under laboratory conditions (12 h day/night cycle; 22–23 °C; humidity, 55–60%) at a ratio of 2:1 with the free access of standard diet and water. On the second day, the female rats were inspected for the presence of a vaginal plug. The day that the vaginal plug was found was counted as day 0 of pregnancy.

The pregnant rats were randomly divided into different groups. RUPP group: The RUPP rat model was established as previously described [13]. Briefly, on gestational day (GD) 14, rats undergoing RUPP procedure were anesthetized with 2% isoflurane (Sigma, St. Louis, MO, USA), and after a midline incision, a 0.203 mm internal diameter silver clip was placed around subrental abdominal aorta. On branches of both ovarian artery, 0.1 mm internal diameter clips were placed above the uterine arteries. Control group: on gestational day 14, the pregnant rats were performed by Sham surgery that have the same abdominal incision and suturing, but don’t have the clip placement. RUPP+Quercetin groups: From GD 14 to GD 21, RUPP rats were performed intragastric administration of different doses of freshly prepared quercetin (Sigma, St. Louis, MO, USA; 10 mg/kg/d, 20 mg/kg/d, 50 mg/kg/d) that was dissolved in 1 mL of 0.9% saline solution. The doses of quercetin used in this study are based on previous reports [14, 15]. RUPP+BQ-123 group: From GD 14 to GD 21, RUPP rats were performed 100 nmol/kg/d BQ-123 (Sigma, St. Louis, MO, USA) treatment through osmotic minipumps.

On day 21 of gestation, the pregnant rats were anaesthetized using isoflurane, fetuses and corresponding placentas were obtained and weighted. Placenta tissues were quick-frozen in liquid nitrogen, then stored at − 80 °C until use. There are no rats were in the process of giving birth on GD21, and the fetuses extracted from the mother. There are 6 rats in each group. All experiments were approved by the Animal Ethics Committee of Qingdao Municipal Hospital (#QDSLYY2018271).

### Blood pressure measure

From GD 13 to GD19, the CODA 6 BP system was used to test blood pressure. Systolic blood pressure (SBP) and diastolic blood pressure (DBP) were tested every 2 days (GD13, GD15, GD17, and GD19). In each session, SBP and DBP were measured for 3 times. On GD21, the rats were anaesthetized, and blood pressure on GD21 was not taken into analysis. The average SBP and DBP were recorded.

### ELISA

Blood of rats on GD21 from different groups was collected and spun at 2500 rpm for 12 min at 4 °C, and plasma was stored at − 80 °C for analysis. The plasma concentrations of ET-1, sFlt-1 and VEGF were detected using commercial ELISA kits according to the manufacturer’s protocols. The sensitivity of the ELISA kits for ET-1, sFlt-1 and VEGF were 0.207 pg/mL, 15.2 pg/mL, 25 pg/mL, respectively. All EILSA kits are purchased from R&D Biosystems (Minneapolis, MN, USA).

### RT-PCR

RNA from placenta tissue on GD21 was extracted by Trizol kit (Thermo Fisher, Waltham, MA, USA). RT-qPCR was carried out using SYBR TaqTM kit (Qiagen, Valencia, CA, USA) in 7300 Real-Time PCR machine (Thermo Fisher, Waltham, MA, USA). The primers used in the study are: preproET-1, Forward: 5′- CTA GGT CTA AGC GAT CCT TGA A-3′ Reverse: 5′- CTT GAT GCT GTT GCT GAT GG-3′; ET_A_R, Forward: 5′-TCA AGC AGC GTC GAG AGG TG-3′, Reverse: 5′-TGT GGC TGC TCC GTT CTG TG-3′; GAPDH, Forward: 5′-AAC TGA GGG CTC TGC TCG CT-3′, Reverse: 5′-GTG ACA CAC CGC AAG GCT T-3′. Ct (cycle threshold) value was collected. The relative expression levels of genes were determined by the 2^−△△Ct^ method.

### Western blotting

On GD21, placental tissue proteins were first extracted, followed by measuring the concentration of the proteins using the BCA kit (Pierce™ BCA, Thermo Fisher Scientific). Proteins were loaded onto a 10% polyvinylidene fluoride membrane (Merck Millipore, Burlington, MA, USA). Then, the samples were blocked using 5% milk for 1 h, followed by incubated using primary antibodies for ET-1 (1:1000, ab2786, Abcam, Shanghai, China) and ET_A_R (1:500, ab85163, Abcam, Shanghai, China) overnight at 4 °C. Horseradish peroxidase-conjugated secondary antibodies were used to incubate temperature for 1 h at room temperature. Finally, chemiluminescent reagents (Merck Millipore, Burlington, MA, USA) were used to detect the signals. Image J was used for quantifying the signals.

### Statistical analysis

SPSS 19.0 was used to analyze all the data. Data were shown as mean ± standard deviation (SD). Kruskal-Wallis test or one-way ANOVA followed with a Tukey post hoc test or two-way ANOVA followed with a Bonferroni post hoc test were used for determining the statistical significance.

## Results

### Quercetin decreased blood pressure during the middle to late gestation period

**Fig. 1:**
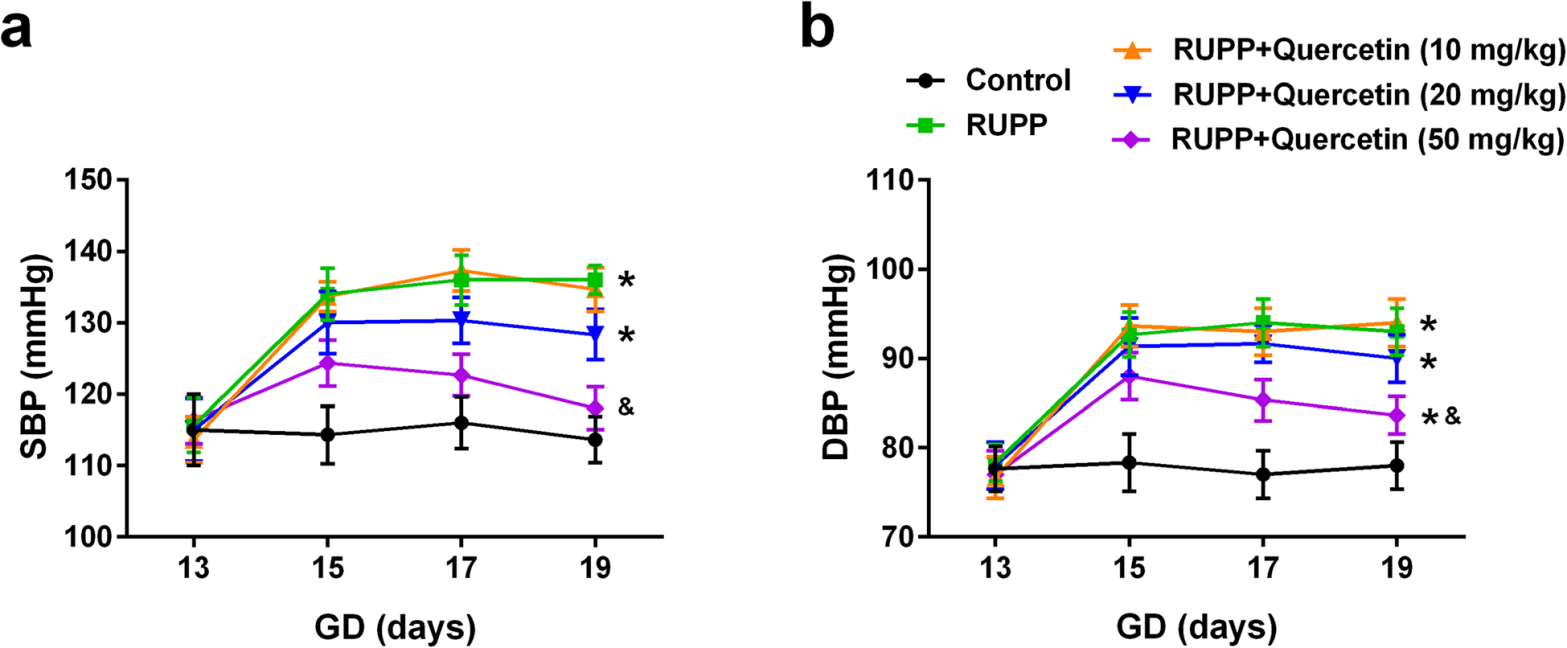
Quercetin decreased systolic (**a**) and diastolic (**b**) blood pressure during the middle to late gestation period. *n* = 6 for each group. Data were shown as mean ± SD. *P < 0.0083 compared with the Control group; ^&^*P* < 0.0083 compared with the RUPP group. Two-way repeated-measures ANOVA with a Bonferroni pos hoc test

The animal model of hypertension in pregnant rats was first established using RUPP. After the surgery on day 14 of gestation, RUPP rats had significantly increased SBP from day 15 to day 19 when compared with control rats. Quercetin treatment by intragastric administration dramatically suppressed SBP of RUPP rats in a dose-dependent manner, as 50 mg/kg quercetin is most effective and decreased SBP from 124.3 ± 3.2 mmHg to 118.0 ± 3.0 mmHg (Fig. 1a). Similarly, Quercetin treatment significantly suppressed diastolic blood pressure (DBP) of RUPP rats from day 15 to day 19 (Fig. 1b). These results demonstrated that quercetin treatment decreased blood pressure during the middle to the late gestation period of RUPP rats.

### Quercetin treatment protects fetal body of RUPP rats

**Fig. 2:**
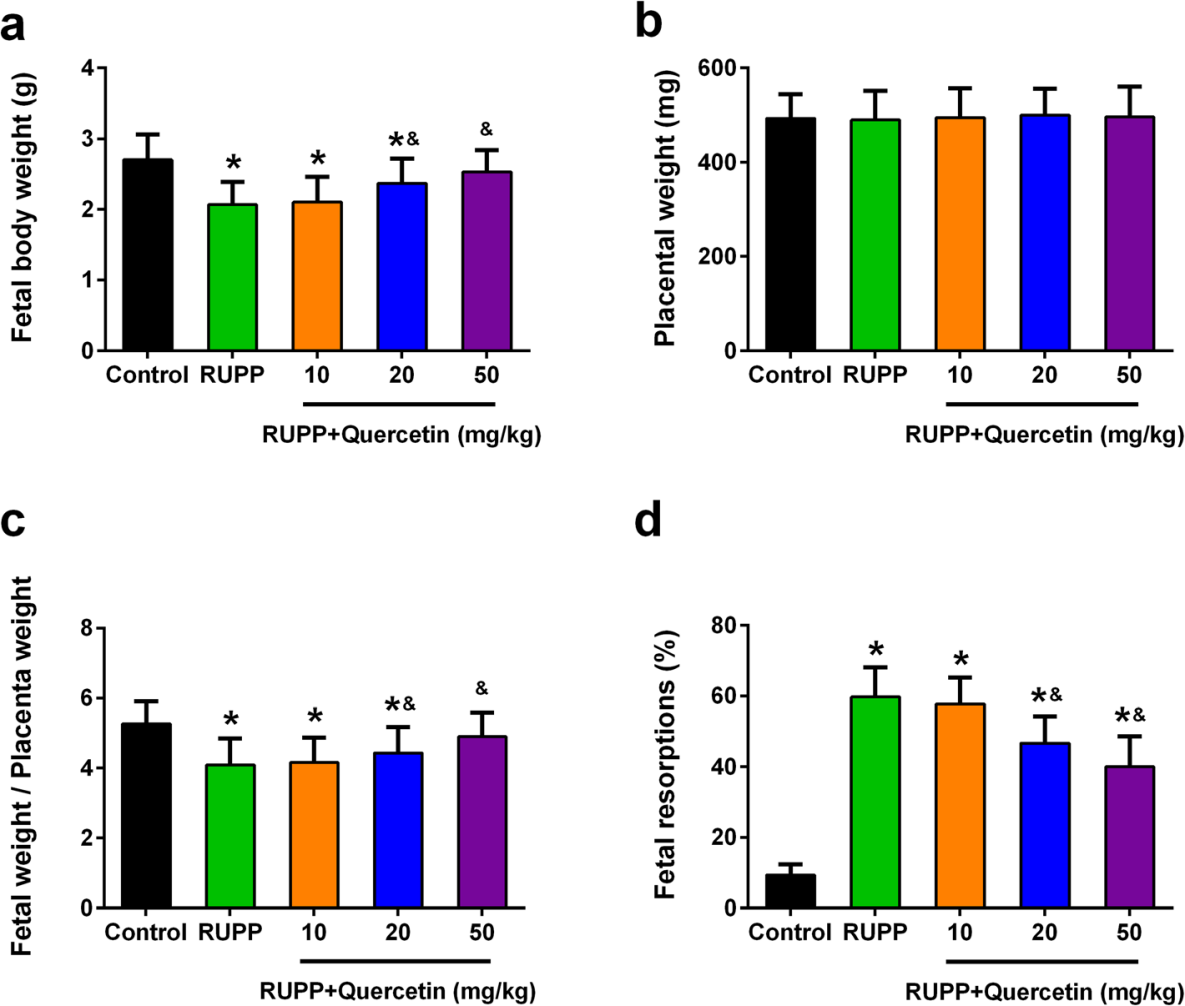
Effects of quercetin administration on features of fetal rats and placenta in each group. Fetal body weight (**a**), placental weight (**b**), fetal/placental weight ratio (**c**) and fetal resorptions (**d**) were determined in pregnant rats at day 21 of gestation. *n* = 55, 24, 30, 36 and 41 in control, RUPP, RUPP+Quercetin (10 mg/kg), RUPP+Quercetin (20 mg/kg) and RUPP+Quercetin (50 mg/kg) group respectively. Data were shown as mean ± SD. *P < 0.05 compared with the Control group; ^&^*P* < 0.05 compared with the RUPP group. One-way ANOVA with a Tukey’s post hoc test (**a**-**c**), Kruskal-Wallis test (**d**)

The effect of quercetin on protecting the fetal body of RUPP rats was further explored. As shown in Fig. 2a, the fetal body weight of RUPP rats (2.07 ± 0.32 g) significantly decreased when compared with the control rats (2.70 ± 0.36 g), but quercetin treatment, especially with high dose dramatically enhanced fetal body weight (2.53 ± 0.31 g). However, quercetin treatment has no effect on placental weight (Fig. 2b). The fetal weight/placenta weight ratio of RUPP rats significantly enhanced after quercetin treatment (Fig. 2c). Moreover, the percentage of fetal resorptions in RUPP rats (59.7 ± 8.4%) was increased as compared with the control group (9.3 ± 3.1%), while high dose quercetin treatment dramatically suppressed the percentage of fetal resorptions in RUPP rats (40.0 ± 8.7%) (Fig. 2d). These results demonstrated that quercetin treatment protects the fetal body of RUPP rats.

### Quercetin treatment attenuates ET-1 and sFLT1, and increased VEGF in plasma of RUPP rats

**Fig. 3:**
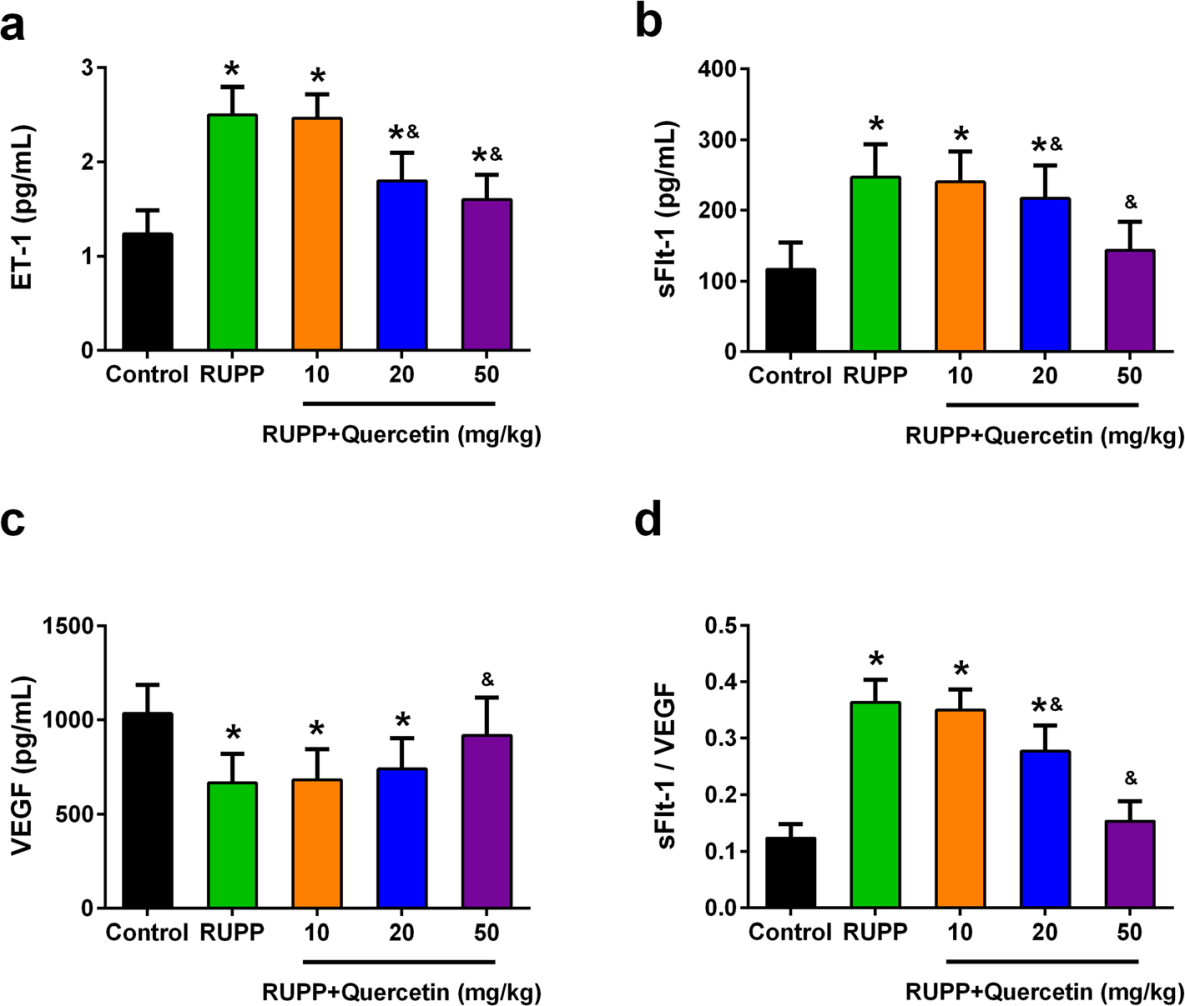
Effects of quercetin on plasma levels of ET-1, sFlt-1 and VEGF of each group at day 21 of gestation. Plasma levels of ET-1 (**a**), sFlt-1 (**b**) and VEGF (**c**) were measured by using ELISA kits. **d** Quercetin administration reduced the sFlt-1/VEGF ratio. *n* = 6 for each group. Data were shown as mean ± SD. *P < 0.05 compared with the Control group; ^&^*P* < 0.05 compared with the RUPP group. One-way ANOVA with a Tukey’s post hoc test

Endothelin-1 (ET-1) was reported to be crucial for preeclampsia pathogenesis and arterial pressure regulation [16–21]. The plasma ET-1 level of RUPP rats (2.51 ± 0.30 pg/mL) was significantly higher when compared with the control rats (1.23 ± 0.25 pg/mL). Quercetin treatment dramatically suppressed the plasma ET-1 level of RUPP rats (1.62 ± 0.27 pg/mL) (Fig. 3a). Circulating fms-like tyrosine kinase-1 (sFlt-1) as well as the VEGF are generally believed to be associated with hypertension in pregnant [22, 23]. The increased plasma level of soluble fms-like tyrosine kinase-1 (sFLT1) in RUPP rats was also inhibited after quercetin treatment (247 ± 47 pg/mL vs. 143 ± 40 pg/mL) (Fig. 3b). As expected, the decreased plasma level of VEGF in RUPP rats was enhanced after quercetin treatment (668 ± 152 pg/mL vs. 916 ± 202 pg/mL) (Fig. 3c). Consistently, the ratio of sFLT1/VEGF in plasma of RUPP rats significantly decreased after quercetin treatment (Fig. 3d). These results demonstrated that quercetin treatment attenuated ET-1 and sFLT1, but increased VEGE in plasma of RUPP rats.

### Quercetin inhibited ET-1, ET_A_R expression of RUPP rats

**Fig. 4:**
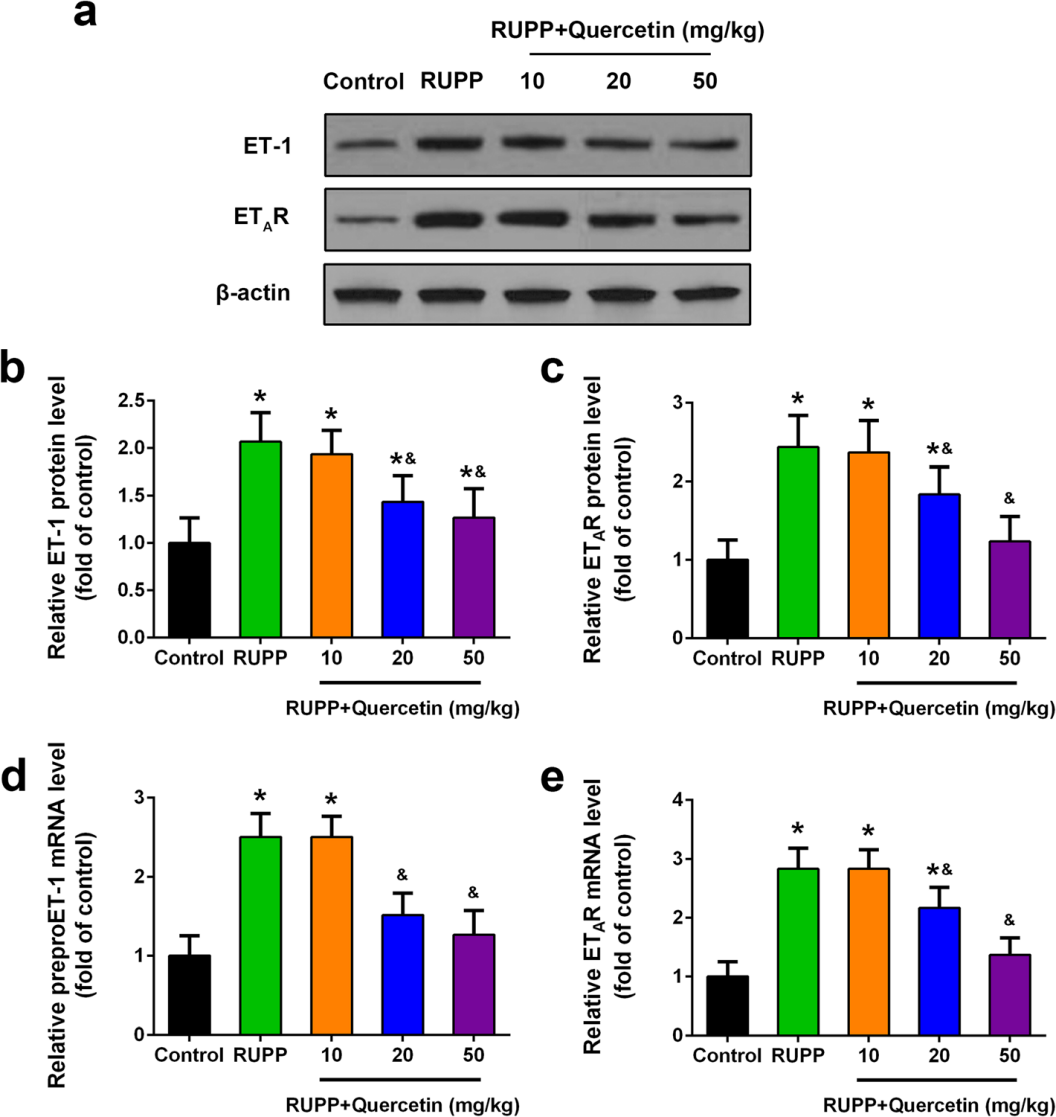
Quercetin inhibited ET-1, ET_A_R expression in placenta tissue of RUPP rats at day 21 of gestation. Western blotting was used to analyze ET-1 and ET_A_R expression (**a**) and relative protein levels were quantified (**b, c**). Relative mRNA levels of preproET-1 (**d**) and ET_A_R (**e**) were detected by qRT-PCR. GAPDH was used as the internal control. *n* = 6 for each group. Data were shown as mean ± SD. *P < 0.05 compared with the Control group; ^&^*P* < 0.05 compared with the RUPP group. One-way ANOVA with a Tukey’s post hoc test

ET_A_R is one of the endothelin receptors (ETR) which were widely believed to be associated with hypertension in pregnancy [24]. The effect of quercetin treatment on ET-1 and ET_A_R expression levels in the placenta tissue of RUPP rats was further detected. On day 21 of gestation, protein expression levels of ET-1 and ET_A_R in the placenta tissue of RUPP rats significantly increased as compared with control rats, while dramatically decreased after quercetin treatment (Fig. 4a to c). Consistently, the mRNA expression levels of ET-1 and ET_A_R significantly increased in the placenta tissue of RUPP rats, while dramatically decreased after quercetin treatment (Fig. 4d and e). These results demonstrated that quercetin inhibited ET-1 and ET_A_R expression of RUPP rats.

### Effects of BQ-123 on blood pressure and outcomes in RUPP rats

**Fig. 5:**
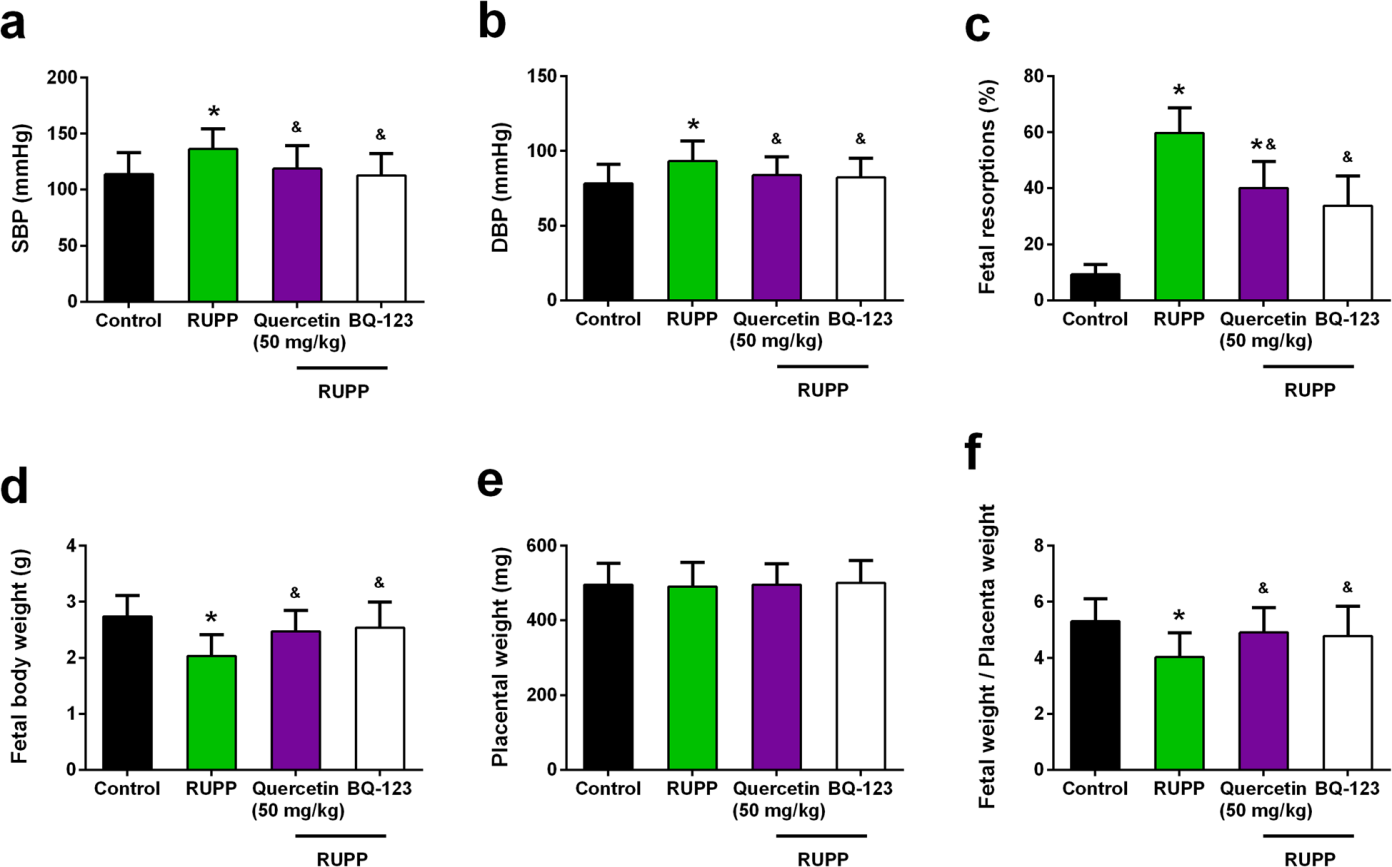
Comparison of the effect of quercetin with the activity of the ET_A_R antagonist BQ-123 on blood pressure and outcomes in RUPP rats. BQ-123 abolishes the increase in systolic (**a**) and diastolic (**b**) blood pressure. Values were measured at day 19 of gestation. Fetal resorptions (**c**), fetal body weight (**d**), placental weight (**e**) and fetal/placental weight ratio (**f**) and were determined in pregnant rats at day 21 of gestation. *n* = 6 for each group in (**a**-**c)**, *n* = 53, 26, 29 and 45 pups in control, RUPP, RUPP+Quercetin (50 mg/kg) and RUPP+BQ-123 group respectively in (**d**-**f)**. Data were shown as mean ± SD. *P < 0.05 compared with the Control group; ^&^*P* < 0.05 compared with the RUPP group. One-way ANOVA with a Tukey’s post hoc test (**a**, **b**, **d**-**f**), Kruskal-Wallis test (**c**)

To further confirm the effect of quercetin on RUPP rats was achieved by inhibiting ET_A_R, the effects of ET_A_R antagonist BQ-123 [25] on blood pressure and outcomes of RUPP rats were determined. Previous study indicated that quercetin has no significant effect on litter size and fetal death of control groups [26]. The procedure and dosage of BQ-123 followed the previous study [25]. BQ-123 administration suppressed systolic (112.3 ± 4.3 mmHg) (Fig. 5a) and diastolic (82.0 ± 3.1 mmHg) (Fig. 5b) blood pressure of RUPP rats. BQ-123 administration suppressed fetal resorptions percentage of RUPP rats (33.7 ± 10.7%) (Fig. 5c), increased fetal body weight (2.53 ± 0.46 g) (Fig. 5d), and showed no influence on placental weight (Fig. 5e). As expected, BQ-123 administration enhanced the ratio of fetal/placental weight (Fig. 5f). The effect of BQ-123 on RUPP rats is similar to the effect of quercetin on RUPP rats.

### Effects of quercetin and BQ-123 on inflammatory cytokines

**Fig. 6:**
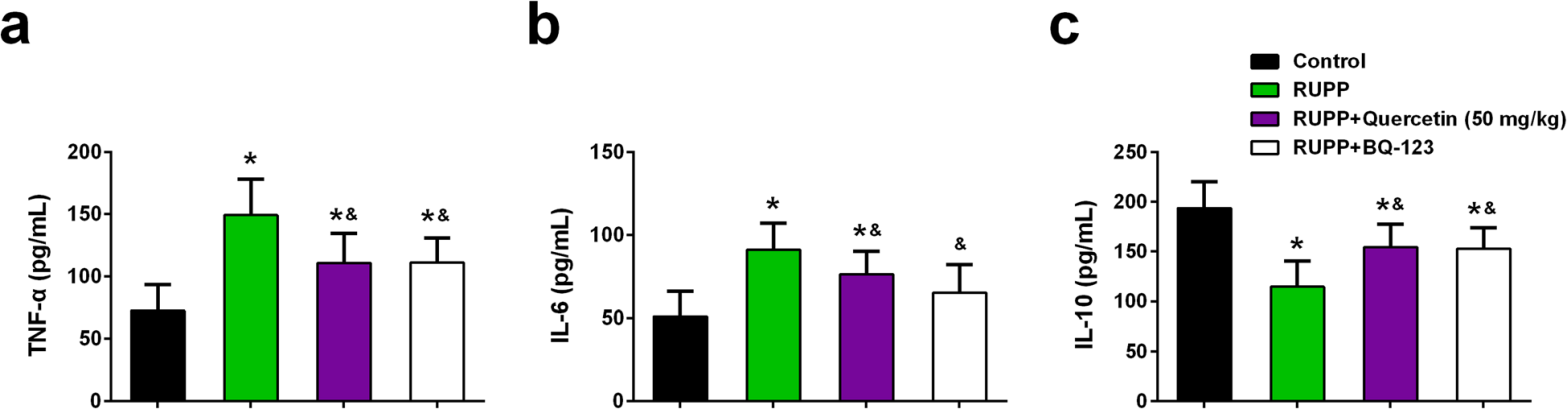
Effects of quercetin and BQ-123 on plasma levels of TNF-α, IL-6 and IL-10 of each group at day 21 of gestation. Plasma levels of TNF-α (**a**), IL-6 (**b**) and IL-10 (**c**) were measured by using ELISA kits. *n* = 6 for each group. Data were shown as mean ± SD. *P < 0.05 compared with the Control group; ^&^*P* < 0.05 compared with the RUPP group. One-way ANOVA with a Tukey’s post hoc test

Quercetin was reported to be crucial for attenuating inflammation. Accordingly, the plasma levels of TNF-α, IL-6, and IL-10 in different groups were determined. The inflammatory cytokine (TNF-α and IL-6) levels of RUPP rats significantly increased when compared with the control rats. Quercetin treatment and the administration of ET_A_R antagonist BQ-123 dramatically suppressed the plasma levels of TNF-α and IL-6 in RUPP rats (Fig. 6a, b).

Oppositely, the plasma level of anti-inflammatory cytokine IL-10 in RUPP rats significantly decreased when compared with the control rats. Quercetin treatment and the administration of ET_A_R antagonist BQ-123 significantly enhanced the plasma level of IL-10 in RUPP rats (Fig. 6c).

## Discussion

Hypertension in pregnancy influences many women, while the treatment for hypertension in pregnancy is limited. Quercetin was reported to be effective to suppress both SBP and DBP in human patients with high blood pressures and in animal models. Accordingly, the effect of quercetin in established hypertension in the pregnant rat model was explored. In RUPP-induced high blood pressure in pregnant rats, different doses of quercetin treatment (10, 20, 50 mg/kg) decreased SBP and DBP, increase fetal body weight, decreased fetal resorptions percentage. Mechanically, quercetin treatment inhibited ET-1/ ET_A_R pathway, and the ET_A_R antagonist suppressed SBP and DBP, and increased fetal body weight, which is similar to the effect of cetin treatment, indicating that quercetin attenuates RUPP induced hypertension in pregnant rats through regulation of ET-1/ ET_A_R pathway.

RUPP was widely used to induce hypertension in pregnant rats. In this study, the pregnant rats were first confirmed. On gestational day 14, the pregnant rats were performed by RUPP as described in methods. Successful hypertension in the pregnant rat model was confirmed by enhanced SBP and DBP of RUPP rats when compared with control rats.

Quercetin was reported to inhibit blood pressure [27]. Patients with type 2 diabetes showed dramatically decreased systolic blood pressure after 10 weeks intake of 500 mg/day quercetin [28]. From one summary of meta-analysis in human data, quercetin intake over 500 mg/day for 8 weeks will benefit patients that have high blood pressure, as evidenced by the decreased SBP and DBP [27]. In animal models of high blood pressure, quercetin also showed the effect to suppress blood pressure. In deoxycorticosterone acetate induced hypertensive model in rats, chronic oral administration of quercetin suppressed blood pressure and improved endothelial dysfunction and aortic dilatation [29]. In spontaneously hypertensive rats, quercetin showed the effect of anti-hypertensive action, enhanced the sensitivity of the baroreflex, suppressed the oxidative stress in serum, indicating that quercetin attenuated blood pressure via reducing oxidative stress [30, 31]. Some other studies reported that quercetin has the ability to destroy renin-angiotensin system through decreasing the expression level and activity of angiotensin converting enzyme [32]. Therefore, the effect of quercetin in anti-hypertensive actions was achieved by regulating vascular compliance, renin-angiotensin system, and the total blood volume. Consistently, quercetin treatment significantly suppressed SBP and DBP of RUPP-induced hypertension rats. More importantly, quercetin treatment improved the outcome of pregnant RUPP rats, as evidenced by increased fetal body weight and decreased fetal resorptions percentage.

ET-1 is expressed by smooth muscle cells and vascular endothelial cells. ET-1 is crucial in regulating vascular functions such as modifying blood flow, altering vessel diameter, and controlling basal arterial tone [33, 34]. ET-1 is effective in blood vessel constriction, and the overexpression of ET-1 always results in high blood pressure. During pregnancy, the effect of ET-1 is enhanced, which will lead to hypertension in pregnancy, even potential heart disease [35, 36]. ET-1 concentration in plasma was reported to be increased in preeclamptic patients that have high blood pressure and be associated with sFlt-1 [37, 38]. Moreover, in animal models of hypertension in pregnancy, the expression levels of ET-1 in kidney and placental increased as compared with normal pregnant rats [39, 40]. All these studies demonstrated that ET-1 may regulate the progression of hypertension in pregnancy. In the present study, ET-1 expression increased in RUPP rats, while quercetin treatment suppressed RUPP expression levels. More importantly, ET_A_R antagonist BQ-123 abolishes SBP and DBP of RUPP rats, which is similar to the effect of quercetin in RUPP rats, demonstrated that the effect of quercetin treatment in RUPP rats is achieved through ET-1/ ET_A_R pathway.

### Study strength and limitations

It was worth noting that there are several limitations for the current study. First, the sample size varied significantly in control, RUPP, RUPP+Quercetin (10 mg/kg), RUPP+Quercetin (20 mg/kg) and RUPP+Quercetin groups, which may lead to false positive results. Second, the conclusion of the involvement of ET-1/ET_A_R pathway in such protective actions of quercetin could be strengthened by employing pharmacological method to active the ET-1/ET_A_R pathway in quercetin-treated RUPP rats, to check whether the activation of the ET-1/ETAR pathway will abolish the protective effect of quercetin on RUPP induced hypertensive rats. Last, although we did not study the side effects of quercetin on pregnancy, embryo, fetus, and placenta, the safety of quercetin could be supported by previous studies. It was reported that quercetin given throughout organogenesis represented no teratogenic threat in the rats [41]. In addition, quercetin treatment did not cause any obvious effects on maternal weight gain, placenta weight, fetal weight and length [26].

## Conclusion

Quercetin attenuates RUPP induced hypertension in pregnant rats through regulation of ET-1 and ET_A_R. Quercetin may be a potential choice for hypertension in pregnancy in the clinic. The effect of quercetin in the clinic should be determined in the future.

## Supplementary information

**Additional file 1.**

## Data Availability

The datasets used or analyzed during the current study are available from the corresponding author on reasonable request.

## Abbreviations

RUPP: Reduced uterine perfusion pressure
SBP: Systolic blood pressure
DBP: Diastolic blood pressure
ET-1: Endothelin-1
sFlt-1: Soluble fms-like tyrosine kinase-1
VEGF: Vascular endothelial growth factor
ELISA: Enzyme-linked immunosorbent assay
ET_A_R: Endothelin-1 type A receptor
GD: Gestational day
