# Supplementary material for: Quercetin attenuates reduced uterine perfusion pressure -induced hypertension in pregnant rats through regulation of endothelin-1 and endothelin-1 type A receptor
Source: Lipids Health Dis. 2020 Aug 5;19:180. doi: 10.1186/s12944-020-01357-w (PMC7409636; doi:10.1186/s12944-020-01357-w)
Supplement: Supplementary file 1 — Additional file 1. [file 12944_2020_1357_MOESM1_ESM.pdf]

Figure 1.

SBP	GD13						GD15						GD17						GD19					
Control	115.8	122.3	120.6	110.4	111.2	109.6	109.8	108.2	117.8	116.6	118.9	114.3	121.4	111.7	119.3	114.5	117.0	112.0	109.2	115.4	109.6	116.0	117.7	114.2
RUPP	120.3	119.7	118.2	112.0	112.8	111.1	128.7	132.1	132.3	136.9	139.9	133.9	130.5	137.4	132.4	138.5	140.7	136.3	134.5	134.0	133.8	137.5	139.3	135.6
RUPP+Quercetin (10 mg/kg)	108.8	114.8	109.8	116.2	117.2	115.3	130.7	132.0	136.7	134.2	135.8	132.7	132.8	133.7	138.5	139.6	140.0	139.1	131.0	132.4	132.0	137.6	139.4	135.7
RUPP+Quercetin (20 mg/kg)	109.7	115.1	108.9	118.7	120.9	116.6	123.8	134.6	123.9	132.5	132.8	132.3	134.2	130.0	135.0	127.5	128.1	126.9	124.7	129.3	127.7	132.9	135.6	130.2
RUPP+Quercetin (50 mg/kg)	112.1	113.3	121.2	117.0	119.2	114.9	128.6	127.5	120.1	123.1	125.5	120.8	119.3	118.3	123.8	124.9	126.3	123.5	114.5	121.7	115.1	118.9	121.8	115.9
DBP																								
Control	74.1	80.6	76.1	78.4	80.9	76.0	77.2	83.2	73.6	78.5	81.2	75.9	81.0	77.1	77.4	75.5	78.4	72.5	79.3	80.6	81.0	75.7	77.5	73.8
RUPP	77.8	74.9	79.7	79.1	81.4	76.7	95.5	95.4	93.3	90.6	92.7	88.5	91.9	94.8	97.9	93.1	96.1	90.1	91.3	96.9	94.2	91.8	94.7	88.9
RUPP+Quercetin (10 mg/kg)	80.1	76.4	73.1	76.8	78.7	74.9	96.4	90.2	95.1	93.5	95.7	91.2	89.4	89.7	93.0	95.3	96.1	94.4	90.2	91.5	93.0	96.4	97.3	95.5
RUPP+Quercetin (20 mg/kg)	80.4	79.9	79.5	76.0	79.0	73.1	94.8	96.1	87.5	89.8	91.3	88.2	94.5	93.3	90.6	90.6	92.9	88.2	86.8	86.5	91.8	91.6	93.5	89.7
RUPP+Quercetin (50 mg/kg)	75.5	73.4	76.5	78.8	81.6	76.1	89.2	89.4	90.9	86.1	89.1	83.1	83.8	85.9	82.2	86.6	89.3	83.9	80.4	84.1	82.0	85.2	86.9	83.4

Figure 2

Fetal body weight (g)

Control	RUPP	RUPP+Quercetin (10 mg/kg)	RUPP+Quercetin (20 mg/kg)	RUPP+Quercetin (50 mg/kg)
2.41	1.91	2.06	2.58	2.28
2.76	1.57	1.89	2.05	2.9
2.55	1.55	1.52	2.43	2.99
2.63	2.34	2.01	2.82	2.43
2.89	1.78	1.71	2.07	2.97
3.1	2.36	2.14	2.75	2.98
2.26	2.28	2.27	2.03	2.46
2.75	2.37	2.49	2.67	2.28
2.67	2.54	1.69	2.42	2.08
2.42	1.91	1.78	2.35	2.07
2.82	2.57	2.53	2.65	2.37
2.31	1.93	2.05	2.81	2.32
2.83	1.82	2.12	1.89	2.83
2.17	2.23	2.29	2.23	2.39
3.17	2.29	1.7	2.8	2.84
2.81	1.96	1.54	2.16	2.82
2.73	2.25	2.57	2.47	2.74
3.21	1.61	2.08	2.69	2.56
2.73	2.29	2.62	1.89	2.83
3.19	1.6	1.68	2.58	2.48
2.42	1.65	1.88	2.82	2.13
3.13	2.28	1.88	2.69	2.66
2.83	2.52	2.3	2.33	2.96
2.38	2.05	2.26	2.52	3
3.14		1.55	2.89	2.07
2.88		2.38	1.97	2.55
2.73		2.46	1.83	2.49
2.14		2.51	2.19	2.2
2.3		2.96	1.86	2.51
2.26		2.06	2.47	2.43
2.76			2.81	2.56
3.25			2.2	2.1
2.56			2.15	2.65
2.63			2.08	2.69
3.24			2.68	2.82
3.14			1.48	2.42
2.71				2.66
2.96				3.01
2.42				2.06

3.06				2.18
2.86				1.94
3.1				
2.87				
2.83				
2.75				
2.67				
3.18				
2.79				
2.87				
2.66				
2.5				
2.61				
1.82				
2.09				
1.54				

placental weight (mg)

Control	RUPP	RUPP+Quercetin (10 mg/kg)	RUPP+Quercetin (20 mg/kg)	RUPP+Quercetin (50 mg/kg)
483.6	482.2	467.4	508.9	464.1
419.2	400.5	481.2	410.8	496.6
430.5	515.2	402.6	460.7	537.3
525.4	501.6	587.3	459.0	541.4
454.6	511.4	477.3	549.9	535.3
498.1	410.2	400.6	587.3	577.9
427.3	431.6	461.5	505.3	472.3
530.3	573.6	434.0	416.2	460.1
494.9	486.1	524.4	437.6	447.9
565.7	550.3	551.9	441.1	476.3
493.3	462.7	540.1	483.9	504.8
533.5	536.6	400.6	492.8	421.5
564.1	474.4	455.7	580.2	441.8
480.4	419.9	528.4	537.4	492.6
417.6	408.2	561.8	439.3	502.7
493.3	523.0	538.2	469.6	523.1
557.6	525.0	471.4	442.9	500.7
441.7	482.2	430.1	539.2	490.6
425.6	406.3	475.3	462.5	445.9
507.7	408.2	420.3	540.9	547.5
428.9	490.0	438.0	571.2	531.2
482.0	586.9	571.6	421.5	561.7
427.3	587.2	581.4	416.2	411.3

502.9	586.6	532.3	500.0	561.7
494.9		430.1	571.2	588.1
494.9		434.0	483.9	545.4
427.3		526.4	434.0	445.9
425.6		575.3	576.6	557.6
515.8		577.9	517.8	559.6
459.4		572.7	521.3	596.2
501.3			569.5	445.9
501.3			542.7	543.4
557.6			435.8	488.5
538.3			557.5	596.2
491.6			558.4	533.2
538.3			556.7	478.4
473.9				553.5
424.0				419.4
528.7				355.6
424.0				356.3
535.1				355.0
478.8				
496.5				
485.2				
496.5				
517.4				
477.2				
443.4				
480.4				
554.4				
446.6				
504.5				
611.0				
611.5				
610.5				

Fetal resorptions (%)

Control	RUPP	RUPP+Quercetin (10 mg/kg)	RUPP+Quercetin (20 mg/kg)	RUPP+Quercetin (50 mg/kg)
5.7	47.3	47.1	37.9	30.2
8.8	69.9	54	36.2	43.3
4.8	71.2	50.7	44.5	26
12.1	56.6	64.8	53.8	46.8
12.4	58	67.3	55	49.4
11.9	55.1	62.2	52.6	44.1

Figure 3.

ET-1 (pg/mL)						
Control	1.32	1.09	1.44	1.17	1.56	0.78
RUPP	2.58	2.38	2.49	2.53	3.04	2.02
RUPP+Quercetin (10 mg/kg)	2.43	2.5	2.36	2.51	2.93	2.08
RUPP+Quercetin (20 mg/kg)	2.03	1.68	1.85	1.74	2.22	1.26
RUPP+Quercetin (50 mg/kg)	1.94	1.6	2.03	1.38	1.44	1.32
sFlt-1 (pg/mL)						
Control	132	176	74	106	107	105
RUPP	310	174	195	267	270	265
RUPP+Quercetin (10 mg/kg)	308	279	169	228	228	228
RUPP+Quercetin (20 mg/kg)	290	274	201	179	181	176
RUPP+Quercetin (50 mg/kg)	177	81	72	176	178	173
VEGF (pg/mL)						
Control	1145	822	1057	1058	1050	1065
RUPP	843	877	429	619	620	618
RUPP+Quercetin (10 mg/kg)	703	574	775	682	682	681
RUPP+Quercetin (20 mg/kg)	808	997	556	693	695	690
RUPP+Quercetin (50 mg/kg)	606	1052	658	1062	1064	1059

Figure 4

preproET-1 mRNA			
Control	1.00	1.21	0.78
RUPP	2.50	2.82	2.17
RUPP+Quercetin (10 mg/kg)	2.50	3.45	1.54
RUPP+Quercetin (20 mg/kg)	1.52	1.75	1.28
RUPP+Quercetin (50 mg/kg)	1.26	1.82	0.69
ETAR mRNA			
Control	1.01	1.23	0.76
RUPP	3.30	2.82	2.35
RUPP+Quercetin (10 mg/kg)	2.83	3.21	2.44
RUPP+Quercetin (20 mg/kg)	2.17	3.28	1.05
RUPP+Quercetin (50 mg/kg)	1.51	1.62	1.39
ET-1 protein			
Control	1.05	1.00	0.94
RUPP	2.06	2.45	1.68
RUPP+Quercetin (10 mg/kg)	1.93	2.23	1.62
RUPP+Quercetin (20 mg/kg)	0.74	2.11	1.43
RUPP+Quercetin (50 mg/kg)	1.27	1.57	0.96
ETAR protein			
Control	0.97	1.02	1.00
RUPP	2.43	3.93	0.93
RUPP+Quercetin (10 mg/kg)	2.37	3.45	1.28
RUPP+Quercetin (20 mg/kg)	1.83	2.73	0.92
RUPP+Quercetin (50 mg/kg)	1.33	1.25	1.10

Figure 5

SBP						
Control	114.3	124	119.8	108	109.8	106.1
RUPP	133.9	138.3	125	139.6	140.8	138.3
RUPP+Quercetin (50 mg/kg)	124.5	103.6	104.3	126.6	128.6	124.5
RUPP+BQ-123	111.3	124.5	122	105.3	106.6	103.9
DBP						
Control	84.2	70.5	75.5	79.2	81.6	76.9
RUPP	96.4	99.2	98.8	87.8	89.8	85.8
RUPP+Quercetin (50 mg/kg)	74.9	90.1	92.8	81.4	81.7	81.1
RUPP+BQ-123	92	76.9	92	77	77.3	76.7
Fetal resorptions (%)						
Control	9	7	11	9.6	11.4	7.7
RUPP	58.9	57	59.2	61	63.1	58.8
RUPP+Quercetin (50 mg/kg)	47.4	46.5	46.5	33.1	34.2	32.1
RUPP+BQ-123	26.1	41.4	28.9	35.2	36.9	33.6

fetal body weight (g)

Control	RUPP	RUPP+Quercetin (50 mg/kg)	RUPP+BQ-123
2.97	2.21	2.64	2.48
2.99	2.26	2.22	2.64
2.54	1.73	2.56	2.17
2.47	2.32	2.28	2.72
2.58	2.13	2.65	2.35
2.7	2.15	2.43	2.19
2.47	2.03	2.53	2.55
2.57	2.23	2.46	2.52
2.82	1.89	2.63	2.5
2.99	2.13	2.35	2.5
2.44	2.1	2.75	2.47
2.83	2.16	2.64	2.57
2.96	1.85	2.7	2.43
3.02	1.83	2.25	2.7
2.92	1.85	2.25	2.8
2.76	2.2	2.47	2.67
2.83	2.15	2.3	2.71
2.76	1.75	2.64	2.44
2.79	2.29	2.42	2.78
2.46	2.32	2.36	2.37
2.91	1.94	2.25	2.61
2.61	2.11	2.32	2.27

2.48	2.13	2.49	2.62
2.77	1.67	2.5	2.81
2.76	1.81	2.71	2.39
2.77	1.53	2.69	2.79
2.52		2.38	2.45
2.46		3.69	2.29
2.56		1.06	2.78
2.64			2.38
2.76			2.37
2.94			2.25
2.54			2.47
2.86			2.59
2.99			2.56
2.76			2.37
2.97			2.44
2.63			2.62
2.47			2.88
2.6			2.57
2.63			2.25
2.62			2.84
2.74			2.56
2.49			3.57
2.82			1.55
2.59			
2.55			
2.86			
2.46			
2.82			
3.08			
4.79			
1.36			

placental weight (mg)

Control	RUPP	RUPP+Quercetin (50 mg/kg)	RUPP+BQ-123
458.9	478.4	462.2	516.3
477.4	474.3	513.1	460.6
487.6	532.9	520.4	520.1
508.8	479.5	533.1	538.3
464.5	448.1	481.3	463.5
463.6	481.6	511.3	476
450.6	481.6	469.5	541.2
505.1	539.1	514	484.6
471.9	469	530.4	515.3
478.3	469	500.4	475

506	494.1	485	482.7
526.3	499.4	455.9	484.6
513.4	525.5	489.5	499
520.8	524.5	478.6	505.7
449.7	524.5	460.4	471.2
498.6	470.1	458.6	490.4
471.9	442.9	534.9	486.5
488.5	463.8	468.6	547
485.7	501.5	495	456.8
507.9	443.9	455.9	523
486.6	518.2	539.5	547
524.5	478.4	496.8	494.2
534.6	486.8	490.4	541.2
474.6	504.3	519.5	517.2
456.2	506.8	490.4	519.1
472.8	501.7	513.1	453.9
489.4		495.7	481.7
494		497.1	488.4
494		494.3	479.8
465.4			489.4
494			457.7
462.6			530.7
475.6			470.2
509.7			480.8
506			520.1
468.2			542.2
538.3			534.5
477.4			496.1
530			547
458.9			464.4
494.9			492.3
474.6			506.7
500.5			502.5
496.8			504.2
466.3			500.8
510.6			
470.9			
473.7			
531.9			
450.6			
605.2			
605.4			
605.1			

Figure 6

TNF-α (pg/mL)						
Control	58.1	67.8	88.3	73.8	74.9	72.6
RUPP	140.9	143.2	156.7	151.6	152	151.2
RUPP+Quercetin (50 mg/kg)	102.8	97.8	129.3	111	113.8	108.1
RUPP+BQ-123	95.9	119.3	125.7	108.3	110.9	105.8
IL-6 (pg/mL)						
Control	50.8	41.9	53	53.6	56	51.2
RUPP	95.8	85.3	99.9	88.3	90.8	85.8
RUPP+Quercetin (50 mg/kg)	79.1	69.9	82	75.4	78.1	72.6
RUPP+BQ-123	59.8	76.9	59.8	64.5	64.7	64.2
IL-10 (pg/mL)						
Control	203.1	191.4	194	191	191.9	190.1
RUPP	130.5	125.1	108.4	108	109.8	106.2
RUPP+Quercetin (50 mg/kg)	140	166.5	166.9	142.9	145.7	164.3
RUPP+BQ-123	149.7	159.4	168.8	146.8	148	145.7
